# A transcription and translation-coupled DNA replication system using rolling-circle replication

**DOI:** 10.1038/srep10404

**Published:** 2015-05-27

**Authors:** Yoshihiro Sakatani, Norikazu Ichihashi, Yasuaki Kazuta, Tetsuya Yomo

**Affiliations:** 1 Department of Bioinformatics Engineering, Graduate School of Information Science and Technology, Osaka University, 1-5 Yamadaoka, Suita, Osaka, 565-0871, Japan; 2 Exploratory Research for Advanced Technology, Japan Science and Technology Agency. 1-5 Yamadaoka, Suita, Osaka, 565-0871, Japan; 3 Graduate School of Frontier Biosciences, Osaka University University, 1-5 Yamadaoka, Suita, Osaka, 565-0871, Japan

## Abstract

All living organisms have a genome replication system in which genomic DNA is replicated by a DNA polymerase translated from mRNA transcribed from the genome. The artificial reconstitution of this genome replication system is a great challenge in *in vitro* synthetic biology. In this study, we attempted to construct a transcription- and translation-coupled DNA replication (TTcDR) system using circular genomic DNA encoding phi29 DNA polymerase and a reconstituted transcription and translation system. In this system, phi29 DNA polymerase was translated from the genome and replicated the genome in a rolling-circle manner. When using a traditional translation system composition, almost no DNA replication was observed, because the tRNA and nucleoside triphosphates included in the translation system significantly inhibited DNA replication. To minimize these inhibitory effects, we optimized the composition of the TTcDR system and improved replication by approximately 100-fold. Using our system, genomic DNA was replicated up to 10 times in 12 hours at 30 °C. This system provides a step toward the *in vitro* construction of an artificial genome replication system, which is a prerequisite for the construction of an artificial cell.

All living organisms have a genomic DNA self-replication system in which genomic DNA is replicated by a DNA polymerase translated from mRNA transcribed from the genomic DNA. Through this transcription- and translation-coupled DNA self-replication, organisms are able to transfer their genetic information to their offspring. Recently, various cellular functions have been artificially constituted in the field of *in vitro* synthetic biology^1^,^2^,^3^,^4^,^5^,^6^, and a plan for genomic DNA replication using rolling-circle-type replication machinery has been proposed^7^,^8^, although it has not yet been realized.

Our group has constructed a single-stranded RNA-based genome replication system coupled with translation^9^ and has observed the autonomous evolution of the RNA genome in a cell-like compartment^10^,^11^. However, all known organisms have DNA-based genomes. To achieve a genome replication system more similar to the natural system, we attempted to construct a transcription- and translation-coupled DNA replication (TTcDR) system.

In this study, we combined the DNA polymerization gene phi29 with a reconstituted *Escherichia coli* translation system to constitute a rolling-circle DNA replication mechanism, following a previous proposal^7^. We found that nucleoside triphosphates (NTPs) and tRNA, which are essential components of the translation system, significantly inhibited DNA replication by phi29 polymerase. By adjusting the concentrations of each of these components, we found conditions under which the initial DNA was replicated up to 10-fold in 12 hours.

## Results

### Scheme for transcription- and translation-coupled DNA replication (TTcDR)

**Figure 1:**
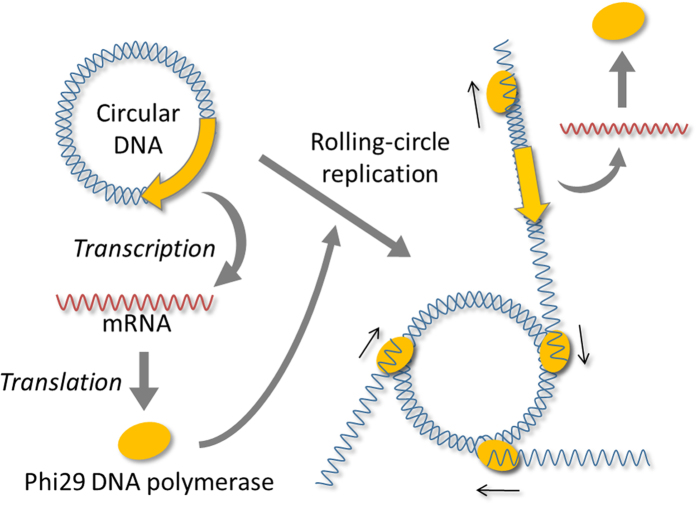
Schematic representation of the transcription- and translation-coupled DNA replication system. Circular DNA encoding phi29 DNA polymerase under control of the T7 promoter is incubated with the reconstituted translation system including T7 RNA polymerase. mRNA is transcribed from the DNA, and phi29 DNA polymerase is translated. The polymerase attaches to the circular DNA and initiates the polymerization of a long single-stranded RNA in a rolling-circle manner. The polymerase further synthesizes the complementary strand to produce double-stranded DNA, which is a long repeat of the circular DNA sequence. The next round of transcription and translation occurs from the double-stranded DNA.

We used circular DNA encoding phi29 DNA polymerase under control of the T7 promoter. The circular DNA was mixed with a reconstituted translation system including T7 RNA polymerase and a random DNA oligo as a primer. First, mRNA was transcribed from the DNA by T7 RNA polymerase, and phi29 DNA polymerase was translated from the mRNA (Fig. 1). Second, the translated phi29 DNA polymerase recognized the oligo DNA attached to the circular DNA and initiated DNA polymerization. Because this polymerase has high processivity and strand-displacement activity, the polymerization continued on the circular template DNA to produce a long single-stranded DNA using the rolling-circle replication process^12^. Third, phi29 DNA polymerase synthesized the complementary strand to produce a long linear repeat of the initial DNA sequence. The final replication product was used as a template for transcription to produce phi29 DNA polymerase for the next round of DNA replication.

### TTcDR reaction

**Figure 2:**
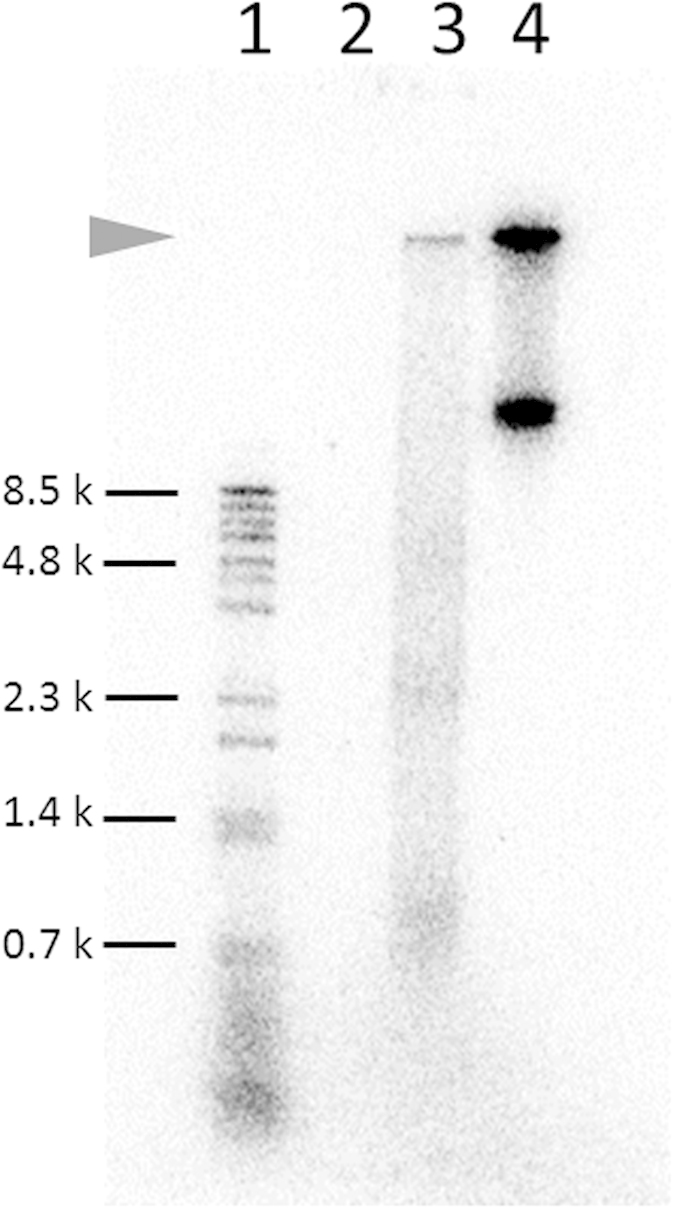
Transcription- and translation-coupled DNA (TTcDR) replication. To perform the TTcDR reaction, circular plasmid DNA encoding phi29 DNA polymerase was incubated with the translation system optimized in a previous study^11^, including dNTPs, yeast ppiase, T7 RNA polymerase, and [^32^P]-dCTP, for 12 h at 30 °C. An aliquot of the mixture after incubation was used in 1% agarose gel electrophoresis and autoradiography. The arrowhead indicates the product of the TTcDR reaction. Lane 1: lambda-BstPI marker. Lane 2: TTcDR reaction without plasmid DNA. Lane 3: TTcDR reaction with plasmid DNA. Lane 4: DNA polymerization with a purified phi29 in phi29 standard buffer.

The circular plasmid DNA encoding phi29 DNA polymerase was mixed with a reaction mixture that included the reconstituted translation system^13^ and T7 RNA polymerase. To perform rolling-circle replication, we also added yeast pyrophosphatase, random hexamers, and dNTPs in a mixture following a previously published method^12^. We incubated this mixture for 12 h at 30 °C with [^32^P]-dCTP to label the synthesized DNA, and after incubation an aliquot of the mixture was applied to agarose gel electrophoresis followed by autoradiography. A band was detected at almost the same position as that of the well (arrowhead in lane 3 of Fig. 2), indicating that the product was very large. Because the product of rolling-circle replication mediated by phi29 polymerase has been reported to be very large (at least 70 kbp)^14^, this result suggests that the detected band is the product of rolling-circle replication, although the amount of product was small (one-tenth of the initial plasmid amount, 1 ng/μl). In a control experiment without the circular plasmid DNA, no band was detected (lane 2). In another control experiment, we performed DNA replication with a purified phi29 polymerase in a standard buffer (lane 4) and found that one of the replication products had the same mobility as the TTcDR product in lane 3, supporting the idea that the TTcDR product was the replication product of phi29 polymerase.

Note that there was another band in lane 4. We do not know why two bands appeared in the replication when using the standard replication buffer, but restriction digestion produced primarily a single band (Fig. S1), indicating that both bands were repeats of the template plasmids. The two bands might represent different states of the replication product, such as an intermediate state with many branching replication forks and the complete single- or double-stranded DNA.

### Optimization of the concentration of the components

**Figure 3:**
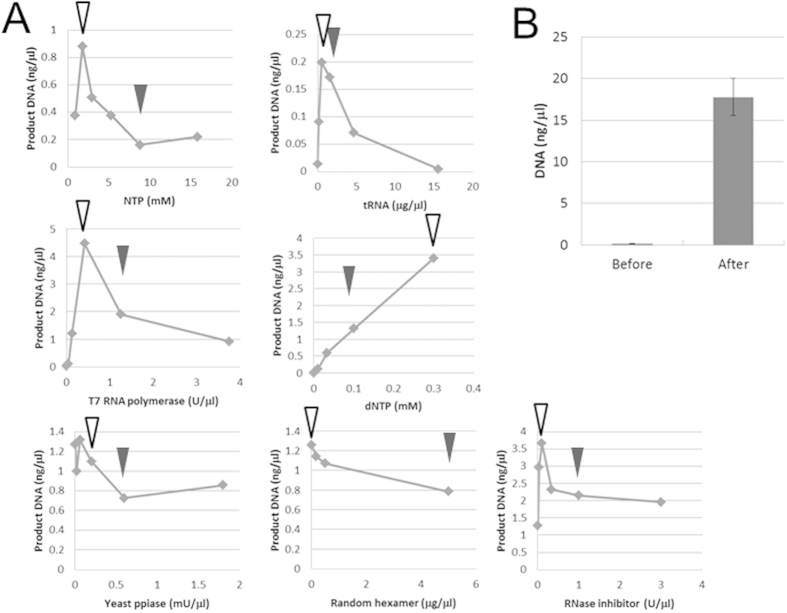
Optimization of the TTcDR system. **A**) Optimization of each component in the TTcDR reaction. TTcDR reactions were performed at the indicated concentrations of the various components with circular DNA (1 ng/μl) for 12 h at 30 °C. The amount of DNA product was measured as described in Fig. 2. The original concentrations and the optimized concentrations are indicated by black and white arrowheads, respectively. NTPs include each nucleotide triphosphate (ATP:GTP:CTP:UTP = 3.75:2.5:1.25:1.25) and the same molarity of magnesium acetate. **B**) Comparison of DNA produced by TTcDR before and after optimization. The TTcDR reaction was performed with 1 ng/μl DNA for 2 h at 30 °C. The compositions before and after optimization are described in the Methods section. The error bars indicate the standard error (n = 14). Original gel images are shown in Figure S2.

One of the difficulties of the TTcDR reaction is performing different types of reactions, e.g., translation and DNA polymerization, in one mixture. We previously experienced this difficulty when constructing an RNA replication system and overcame it by optimizing the concentration of components^11^. Applying the same strategy to DNA replication, we attempted to optimize the concentrations of several components in the system. We changed the concentrations of NTPs, tRNA, T7 RNA polymerase, the random DNA oligo hexamers, the yeast pyrophosphate (ppiase), dNTPs, and the RNase inhibitor, and we measured the amount of replication products after the TTcDR reaction (Fig. 3A).

The concentration dependencies of NTPs, tRNA, T7RNA polymerase, and RNase inhibitor, showed sharp bell-shaped curves, indicating that the optimum concentrations existed in narrow ranges. The amount of replication product showed an almost linear increase that was dependent on dNTP concentration up to 3 mM. The replication product was relatively insensitive to the yeast ppiase and random hexamer concentrations; the replication product did not change more than 2-fold over the examined concentration ranges. Unexpectedly, we observed DNA replication in the absence of the random hexamers, although the random hexamers were expected to be necessary for the initiation of DNA replication in this system. A possible mechanism is examined later. At optimized concentrations of all components, the amount of replication product increased by approximately 100-fold compared to original conditions (Fig. 3B) and reached approximately 10 times the amount of the initial plasmid DNA after 10 h of incubation at 30 °C.

The bell-shaped curves for tRNA, NTPs, and T7 RNA polymerase indicated that these compounds inhibited certain steps of the TTcDR reaction. These compounds are necessary for translation and have no inhibitory effects on translation even at high concentrations^15^; therefore, we tested the possibility that these components inhibited DNA polymerization by phi29 polymerase. We measured the effects of these compounds on DNA polymerization by purified phi29 DNA polymerase in standard replication buffer. tRNA and NTPs significantly inhibited DNA replication, whereas T7 RNA polymerase had only a small effect (Fig. S3), indicating that tRNA and NTPs were strong inhibitory factors for DNA polymerization by phi29 polymerase.

### Characterization of the TTcDR reaction and product

**Figure 4:**
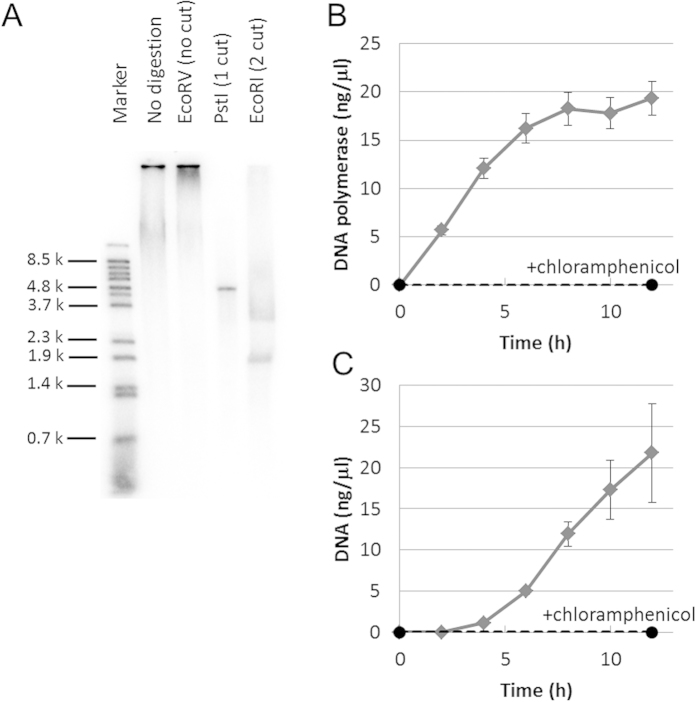
Characterization of the optimized TTcDR reaction. **A**) Cleavage of the TTcDR product by restriction enzymes. After the TTcDR reaction was conducted for 12 h at 30 °C, the indicated restriction enzymes were added and incubated for 1 h at 37 °C. An aliquot was used for 1% agarose gel electrophoresis and autoradiography. The sample treated with *Pst*I was purified using a DNA column (Life Technologies) before electrophoresis. **B**) Time-course data for the translation of DNA polymerase during the TTcDR reaction. The circular DNA (1 ng/μl) was incubated with the optimized TTcDR mixture containing [^35^S]-methionine at 30 °C with or without chloramphenicol (25 μg/μl). After the indicated time, an aliquot was used for 10% SDS-PAGE and autoradiography. The error bars indicate the standard error (n = 4). **C**) Time-course data for DNA replication during the TTcDR reaction. The circular DNA (1 ng/μl) was incubated with the optimized TTcDR mixture containing [^32^P]-dCTP at 30 °C with or without chloramphenicol (25 μg/μl). After the indicated time, an aliquot was used for 1% agarose gel electrophoresis and autoradiography. The error bars indicate the standard error (n = 4).

We characterized the replication product of the optimized TTcDR reaction. First, we digested the TTcDR product with the restriction enzymes *Eco*RV (0 cuts in the initial plasmid), *Pst*I (1 cut in the initial plasmid), and *Eco*RI (2 cuts in the initial plasmid). If the TTcDR product was a repeat of the plasmid sequence expected by rolling-circle replication, *Pst*I or *Eco*RI treatment should produce a 4.7 kbp or 1.9 + 2.8 kbp DNA, respectively, whereas EcoRV treatment should have no effect on the band pattern. As expected, most of the TTcDR product shifted to 4.7 kbp or 1.9 + 2.8 kbp after *Pst*I or *Eco*RI treatment, respectively, whereas *Eco*RV treatment had no effect (Fig. 4A). Even after *Pst*I or *Eco*RI treatment, some TTcDR product with the same mobility remained, which may have been a nonspecific replication product generated from contamination with circular DNA, as often reported in rolling-circle replication^16^,^17^,^18^,^19^. To further examine the replication product, we performed PCR using the replication product as a template and primers specific to the original plasmid, and found that DNA of the expected size (4.7 kbp) was amplified only when the TTcDR product after incubation was used as a template, but not when the mixture before incubation was used as a template (Fig. S5). Sequence analysis of approximately 500 bp from both termini revealed that the amplified DNA was the same as the original plasmid sequence.

We next obtained time-course data on translation and replication in the TTcDR reaction. The translation of phi29 DNA polymerase increased linearly until 6 h and then became almost constant (Fig. 4B). This increase was not observed in the presence of chloramphenicol, an inhibitor of translation. DNA replication showed a concave curve until 12 h, and the concentration of the product was higher than the initial plasmid concentration (1 ng/μl) after 6 h (Fig. 4C). This DNA replication was also not observed in the presence of chloramphenicol. The concave curve of the DNA replication indicates that replication accelerated during the reaction, consistent with the expected behavior of the TTcDR reaction, in which the amount of DNA polymerase increases over time, thereby accelerating DNA replication.

### Further transcription and translation from the TTcDR product

**Figure 5:**
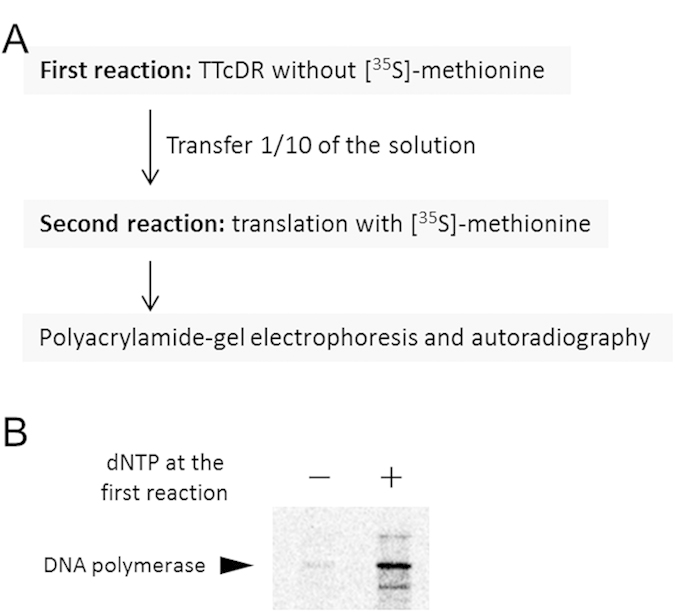
Translation of phi29 DNA polymerase from newly synthesized DNA in the TTcDR reaction. **A**) Experimental procedure. First, we performed the optimized TTcDR reaction without [^35^S]-methionine in the presence or absence of dNTPs, and one-tenth of the mixture was transferred to the second reaction mixture, which contained [^35^S]-methionine, to detect translation from the replicated DNA product in the first reaction. After incubation at 30 °C for 12 h, an aliquot was used for 10% SDS-PAGE and autoradiography. **B**) Translation results. Increased translation of the DNA polymerase was detected when the first reaction contained dNTPs, indicating that the translation occurred from the DNA produced in the first reaction.

We tested whether the TTcDR product could serve as a template for the next round of transcription and translation, which is one of the requirements for continuous DNA replication. The experimental procedure is shown in Fig. 5A. First, we performed the TTcDR reaction without [^35^S]-methionine for 12 h at 30 °C. As a control experiment, we also performed the first reaction in the absence of dNTPs to inhibit DNA replication. Then, an aliquot (1/10) from each first reaction was transferred to a second reaction mixture containing a fresh translation system and [^35^S]-methionine. After further incubation for 12 h at 30 °C, an aliquot was applied to SDS-PAGE followed by autoradiography to detect the translated polymerase in the second reaction. A larger amount of the translated polymerase was detected when the first reaction was performed in the presence of dNTPs than in the absence of dNTPs, indicating that the replicated DNA from the first reaction functioned as a template for transcription and translation in the second reaction (Fig. 5B).

### A possible mechanism for TTcDR without random hexamers

**Figure 6:**
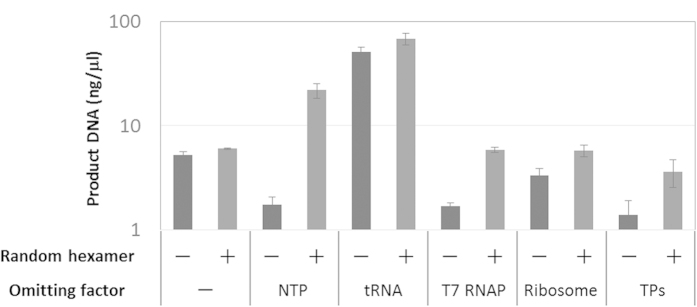
DNA replication with or without random hexamers in the absence of TTcDR components. DNA replication was performed by purified phi29 DNA polymerase with or without random hexamers in the TTcDR mixtures in which some of the components (NTP, tRNA, T7 polymerase, ribosome, and translation proteins) were omitted, and the amount of replicated DNA was measured as described in the Methods section. The translation proteins contained all protein factors in the translation system (e.g., IFs, EFs, RFs, and aminoacyl-tRNA synthetases). In the experiments with random hexamers, the template plasmid was heated with the hexamers at 95 °C for 3 min and then cooled immediately.

The TTcDR system constructed in this study did not require the addition of random hexamers or heating for hybridization, which were required for DNA polymerization by phi29 polymerase in previous studies^12^,^20^,^21^. One possible explanation is that the RNA produced by transcription served as a primer because RNA is processed by phi29 polymerase and can function as a primer^22^. To test this hypothesis, we performed DNA replication with purified phi29 polymerase in the absence of one component of the TTcDR system (NTP, tRNA, ribosome, T7 polymerase, or translation proteins (TPs)) with or without the random hexamers (Fig. 6). We found that DNA replication without the random hexamers significantly decreased in the absence of T7 polymerase or NTP, supporting the hypothesis that RNA transcribed by T7 polymerase served as a primer. However, a certain amount of DNA replication occurred even without T7 polymerase or NTP, and DNA replication without random hexamers significantly decreased in the absence of translation proteins. These results suggest that there is a mechanism to start replication in the absence of random hexamers.

## Discussion

In this study, we constructed a DNA replication system using a rolling-circle mechanism coupled with transcription and translation. This type of replication was proposed approximately 8 years ago by Forster and Church^7^,^8^, but it had not yet been realized, possibly because of the large inhibitory effect of NTPs and tRNA, required for translation, on DNA replication (Fig. S3). This characteristic, that components required for one function inhibit another function, makes it difficult to combine different types of reactions in one vessel. To circumvent this difficulty, we searched for the optimum concentrations to adjust the system to an optimum balance between translation and DNA replication, and we achieved approximately 10-fold DNA amplification in 12 h at 30 °C. This replication system represents a step toward the realization of a DNA-based genome replication system, which is necessary for the synthesis of an artificial cell.

A significant shortcoming of this TTcDR system involves recursiveness; the initial template DNA is circular but the product is a long linear concatemer. To make this system recursive, the linear DNA product must be circularized. In a previous proposal, the product was circularized through DNA recombination by Cre recombinase^23^. We attempted to use this method, but the recombination product was not detected, probably because of low recombination efficiency (data not shown). The low efficiency may have arisen because the optimal conditions for recombination are different from those for translation or DNA replication. Determination of conditions under which all reactions occur sufficiently will be an important subsequent challenge.

NTPs and tRNA significantly inhibited DNA polymerization by phi29 DNA polymerase at physiological concentrations (Fig. S3). These inhibitory effects are counterintuitive because phi29 DNA polymerase functions physiologically in cells, which contain abundant NTPs and tRNA^24^. One possible explanation for these inhibitory effects could be an unknown regulation mechanism acting on the polymerase during infection by the phi29 bacteriophage. For instance, inhibition by NTPs may be useful to temporally separate mRNA transcription and DNA polymerization because both reactions use the same DNA as a template and often collide^25^; when NTPs are abundant, RNA transcription occurs but DNA polymerization is inhibited, and after NTPs are consumed, DNA polymerization begins. Although further investigation is required to test this hypothesis, the characterization of phi29 DNA polymerase in this study provides useful information regarding the replication of the phi29 bacteriophage.

## Methods

### Plasmid construction

The template plasmid used in all experiments (pUC-phi29DNAP-loxP) was constructed as follows. First, we introduced a DNA fragment encoding the phi29 DNA polymerase gene into a pET vector to produce pET-phi29-DNAP. This plasmid was constructed by ligation of a PCR fragment prepared with the primers GGAGATATACATATGCCGAGAAAGATGTATAGTTGTG and AGCTCGAATTCATTATTTGATTGTGAATGTGTCATCAAC using the phi29 phage genome as a template and another PCR fragment prepared with the primers TAATGAATTCGAGCTCCGTCG and CATATGTATATCTCCTTCTTAAAGTTAAAC using pET21 as a template and an InFusion kit (Clontech). We next changed the vector from pET to pUC to reduce the plasmid size, which is an important factor for replication. Using pET-phi29-DNAP as a template, we amplified a DNA fragment including the DNA polymerase gene, ribosome-binding site, and T7 promoter with the primers AAAAGCATGCTAATACGACTCACTATAGGG and AAAAGCATGCGCTAGTTATTGCTCAGCGG and ligated it to the *Sph*I site of pUC19 after *Sph*I cleavage to produce pUC-phi29DNAP. Next, we introduced a loxP sequence into this plasmid for future use by amplifying the whole sequence with the primers ATAGCATACATTATACGAAGTTATCCGCTGAGCAATAACTAGCG and TATAATGTATGCTATACGAAGTTATTGGCAGCAGCCAACTCAGC, which contained the loxP sequence, and self-ligated the plasmid with the Infusion kit to produce the template plasmid (pUCphi29DNAP-loxP). The phi29 phage was kindly provided by Osamu Makino of Sophia University, Japan.

### Assay of the TTcDR reaction

The optimized composition of the TTcDR system was as follows: template plasmid DNA (1 ng/μl), dNTPs (0.3 mM each, Takara), [^32^-P] dCTP (3.3 μM, PerkinElmer), magnesium acetate (7.9 mM, Wako), potassium glutamate (70 mM, Wako), spermidine (0.375 mM, Nakarai), dithiothreitol (6 mM, Nakarai), ATP (0.375 mM, GE Healthcare), GTP (0.25 mM, GE Healthcare), CTP (0.125 mM, GE Healthcare), UTP (0.125 mM, GE Healthcare), creatine phosphate (25 mM, Nakarai), *E. coli* tRNA mixture (0.518 μg/μl, Roche), 10-formyl-5,6,7,8-tetrahydrofolic acid (10 ng/μl), Cys (0.3 mM, Wako), Tyr (0.3 mM, Wako), the other 18 amino acids except for Cys and Tyr (0.36 mM, Wako), 2-[4-(2-hydroxyethyl)-1-piperazinyl]ethanesulfonic acid (100 mM, pH 7.6, Sigma), ribosomes (1 μM), IF1 (25 μM), IF2 (1 μM), IF3 (4.9 μM), EF-G (1.1 μM), EF-Tu (80 μM), EF-Ts (3.3 μM), RF1 (0.05 μM), RF2 (0.05 μM), RF3 (0.17 μM), RRF (3.9 μM), AlaRS (730 nM), ArgRS (30 nM), AsnRS (420 nM), AspRS (120 nM), CysRS (20 nM), GlnRS (60 nM), GluRS (230 nM), GlyRS (90 nM), HisRS (90 nM), IleRS (370 nM), LeuRS (40 nM), LysRS (120 nM), MetRS (110 nM), PheRS (130 nM), ProRS (170 nM), SerRS (80 nM), ThrRS (80 nM), TrpRS (30 nM), TyrRS (150 nM), ValRS (20 nM), MTF (590 nM), creatine kinase (0.25 μM), myokinase (1.4 μM), nucleoside-diphosphate kinase (20 nM), pyrophosphatase (40 nM), yeast inorganic pyrophosphatase (0.2 mU/μl, New England BioLabs (NEB)), ribonuclease inhibitor (0.1 U/μl; Promega), and T7 RNA polymerase (0.42 U/μl; Takara). Most of the proteins described above were purified according to a previously described method^11^ except for yeast inorganic pyrophosphatase, ribonuclease inhibitor, and T7 RNA polymerase. The original composition differed in the concentrations of dNTPs (0.1 mM each), yeast inorganic pyrophosphatase (0.6 mU/μl), T7 RNA polymerase (1.25 U/μl), ribonuclease inhibitor (1 U/μl), ATP (3.75 mM), GTP(2.5 mM), CTP (1.25 mM), UTP (1.25 mM), *E. coli* tRNA mixture (1.55 μg/μl), magnesium acetate (16 mM), and random hexamers (5 μM NEB).

The reaction mixture was incubated at 30 °C for 12 h and applied to 1% agarose gel electrophoresis. After fixation of the gel in a solution containing 10% methanol and 10% acetic acid, the gel was dried and subjected to autoradiography (FLA 7000, GE Healthcare). The band intensity of the TTcDR product was measured by software (Image Quant, GE Healthcare) and compared to that of a spot of the reaction mixture before incubation that contained a known concentration of total dCTP, almost the same as the total unlabeled dCTP (0.3 mM) because the labeled [^32^P]-dCTP amount was negligible. From the comparison, we determined the amount of DNA contained in each band, and also the original concentrations in the mixture.

### Assay of translation

The TTcDR reaction mixture containing [^35^S]-methionine instead of [^32^P]-dCTP was incubated at 30 °C, and aliquots were applied to 10% SDS-PAGE. After fixation of the gel in a solution containing 10% methanol and 10% acetic acid, the gel was dried and subjected to autoradiography (FLA 7000, GE Healthcare). The band corresponding to the phi29 DNA polymerase was quantified in the same manner as for DNA quantification.

### Assay of DNA replication by purified phi29 DNA polymerase in a standard buffer

The standard reaction buffer contained the template plasmid DNA (1 ng/μl), dNTPs (0.3 mM each), [^32^P]-dCTP (3.3 μM), phi29 Tris-HCl (50 mM, pH 7.8), magnesium chloride (5 mM), potassium chloride (7.5 mM), dithiothreitol (0.1 mM), and purified phi29 DNA polymerase (1 U/μl, NEB). In the experiment shown in Figure S3, the indicated concentration of NTP mixture (ATP:GTP:CTP:UTP:magnesium acetate = 3:2:1:1:7.2), *E. coli* tRNA mixture, or T7 RNA polymerase was added. The mixture was incubated at 30 °C for 12 h and applied to agarose gel electrophoresis as described above.

## Additional Information

**How to cite this article**: Sakatani, Y. *et al.* A transcription and translation-coupled DNA replication system using rolling-circle replication. *Sci. Rep.* **5**, 10404; doi: 10.1038/srep10404 (2015).

## Supplementary Material

Supplementary Information
