# Supplementary material for: A transcription and translation-coupled DNA replication system using rolling-circle replication
Source: Sci Rep. 2015 May 27;5:10404. doi: 10.1038/srep10404 (PMC4445062; doi:10.1038/srep10404)
Supplement: Supplementary Information [file srep10404-s1.pdf]

Supplementary Information

A transcription and translation-coupled DNA replication system using rolling-circle replication

Yoshihiro Sakatani[a], Norikazu Ichihashi[a, b], Yasuaki Kazuta[b], Tetsuya Yomo*[a, b, c, d]

[a] *Department of Bioinformatics Engineering, Graduate School of Information Science and Technology, Osaka University, 1-5 Yamadaoka, Suita, Osaka, 565-0871, Japan*

[b] Exploratory Research for Advanced Technology, Japan Science and Technology Agency. 1-5 Yamadaoka, Suita, Osaka, 565-0871, Japan

[c] Graduate School of Frontier Biosciences, Osaka University University 1-5 Yamadaoka, Suita, Osaka, 565-0871, Japan

[d] Earth-Life Science Institute, Tokyo Institute of Technology, Meguro, Tokyo, 152-8550, Japan

*yomo@ist.osaka-u.ac.jp

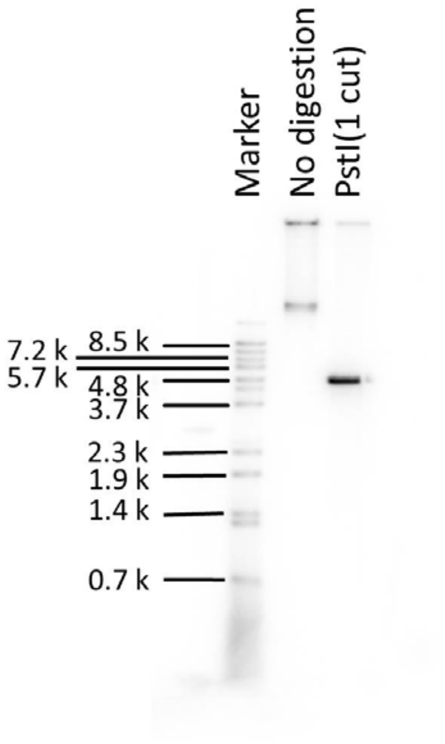

Figure S1 Digestion of the replication product in standard replication buffer.
The DNA replication product produced by purified phi29 DNA polymerase in the buffer system shown in lane 4 of Figure 2 was digested with PstI, which cleaved the template plasmid (4.7 kbp) at one location.

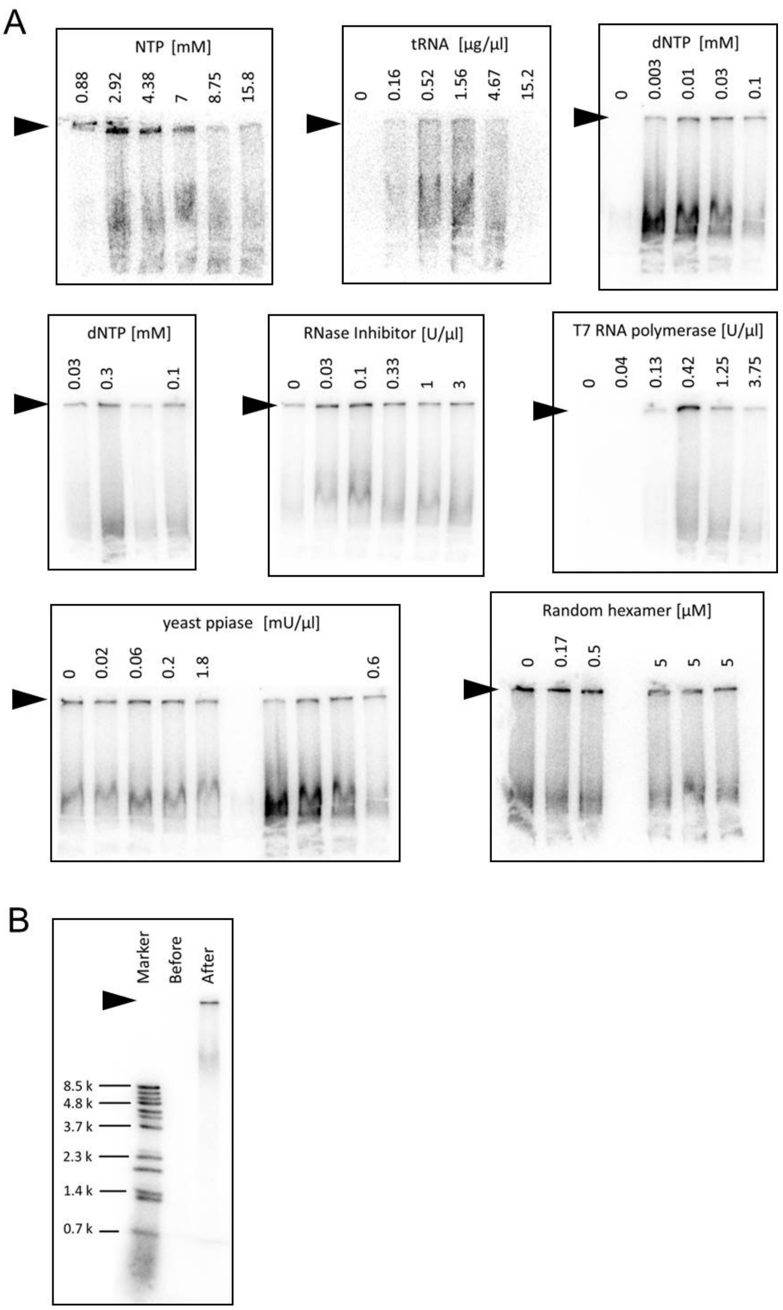

Figure S2 Whole gel images of Figure 3.

(A) Whole gel images of Figure 3A. (B) One of the whole gel images of Figure 3B. The quantified bands are indicated (arrowheads).

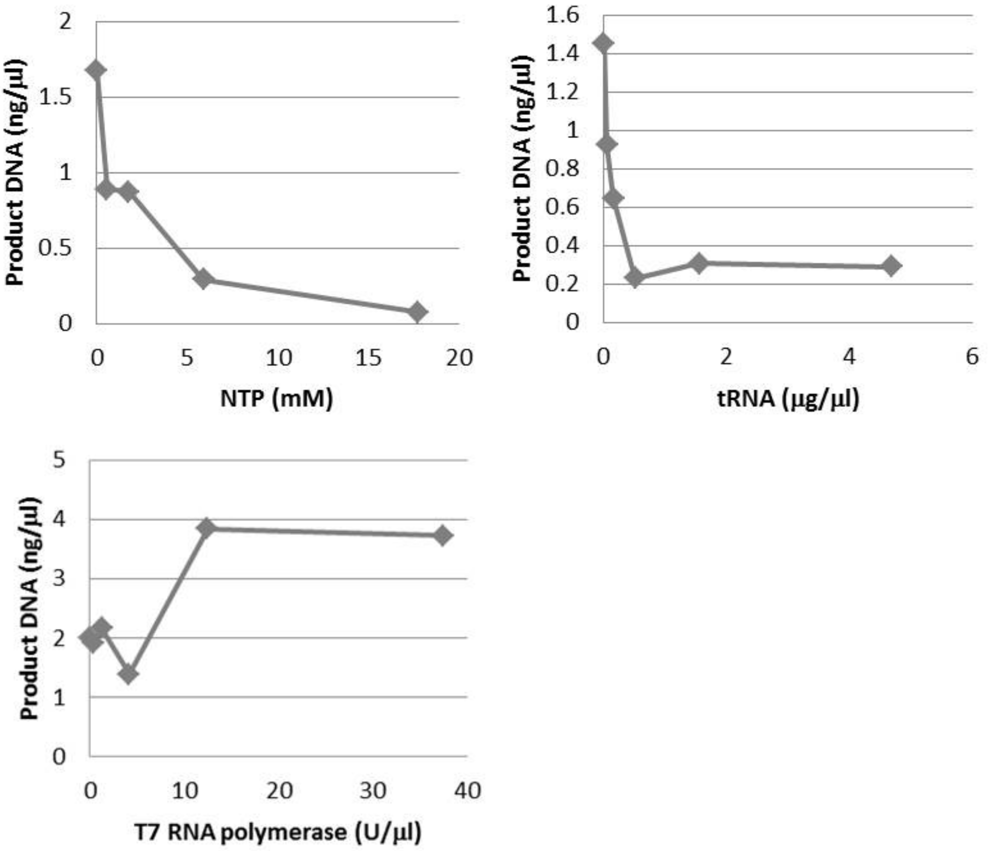

Figure S3 Effects of certain components on DNA replication

Circular DNA (1 ng/μl) was mixed with purified phi29 DNA polymerase (1 U/μl) in standard replication buffer and incubated at 30 °C for 12 h in the presence of NTPs, tRNA, or T7 RNA polymerase at the indicated concentrations. The replication products were measured as described in the Methods. NTPs included each nucleotide triphosphate (ATP:GTP:CTP:UTP = 3.75:2.5:1.25:1.25) and the same molarity of magnesium acetate. Original gel images are shown in Figure S4.

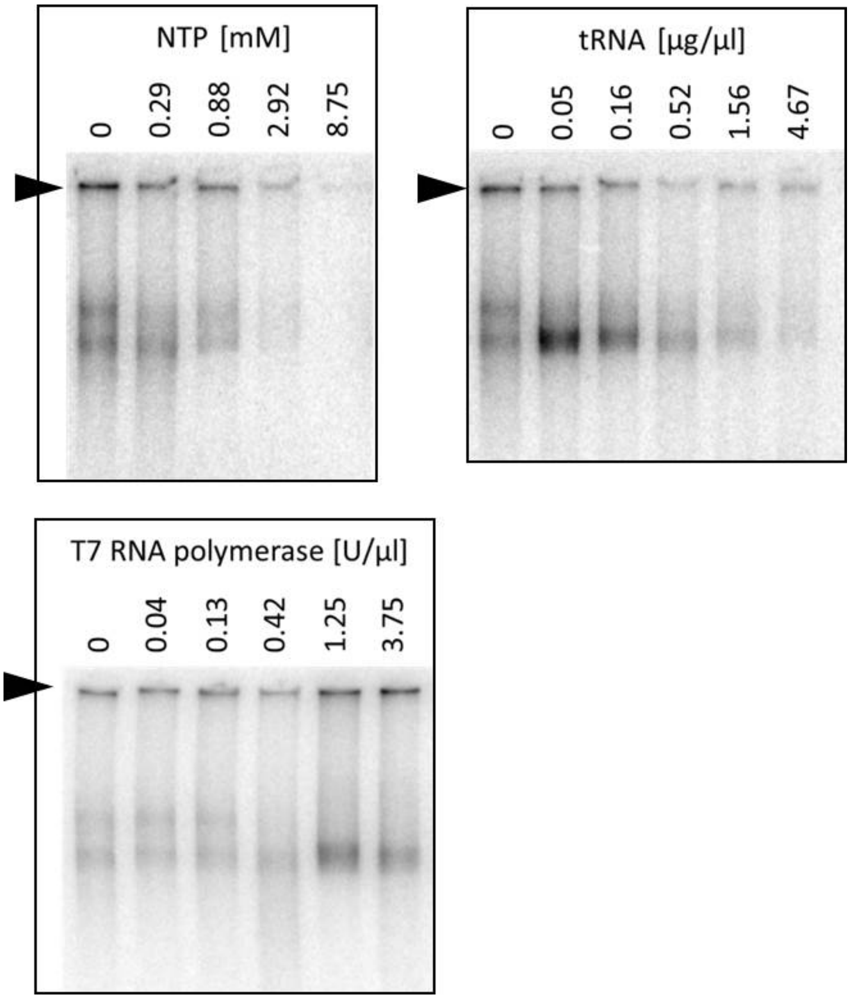

Figure S4 Whole gel images of Figure S3

The quantified bands are indicated (arrowheads).

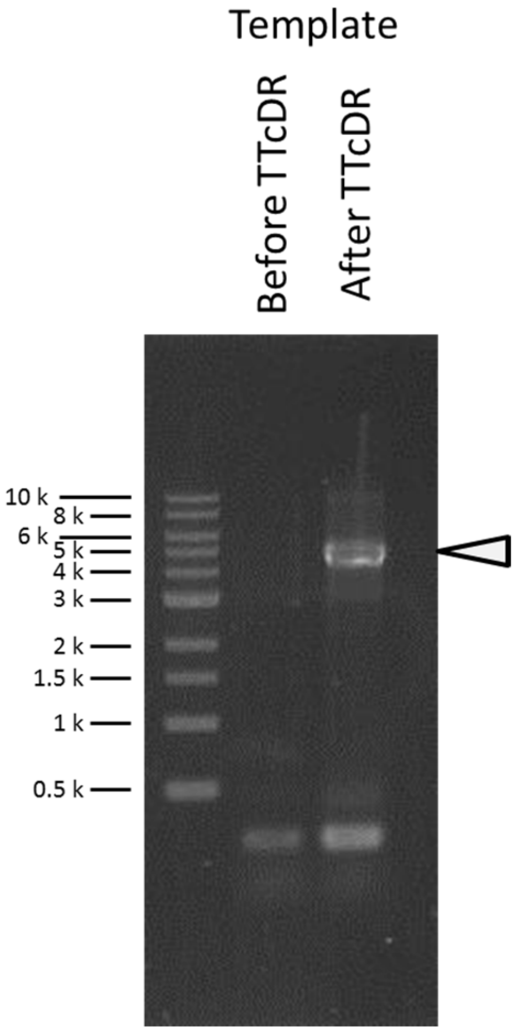

Figure S5 **Amplification of the plasmid sequence from the TTcDR product by PCR.**
We performed PCR using the TTcDR product after a 12 h incubation as a template and specific primers for the original plasmid (GTTATCCACAGAATCAGGGGATAAC and CGTATTACCGCCTTTGAGTGAGC). As a control, we also used the TTcDR mixture before incubation as a template. To avoid amplifying from the original plasmid, we treated the TTcDR mixture with *Dpn*I (final 0.5U/μl) at 37 °C for 2 h to degrade the template plasmid. If the TTcDR product was a repeat of the original plasmid, a 4.7 kbp DNA fragment (arrowhead) would be amplified by this PCR.

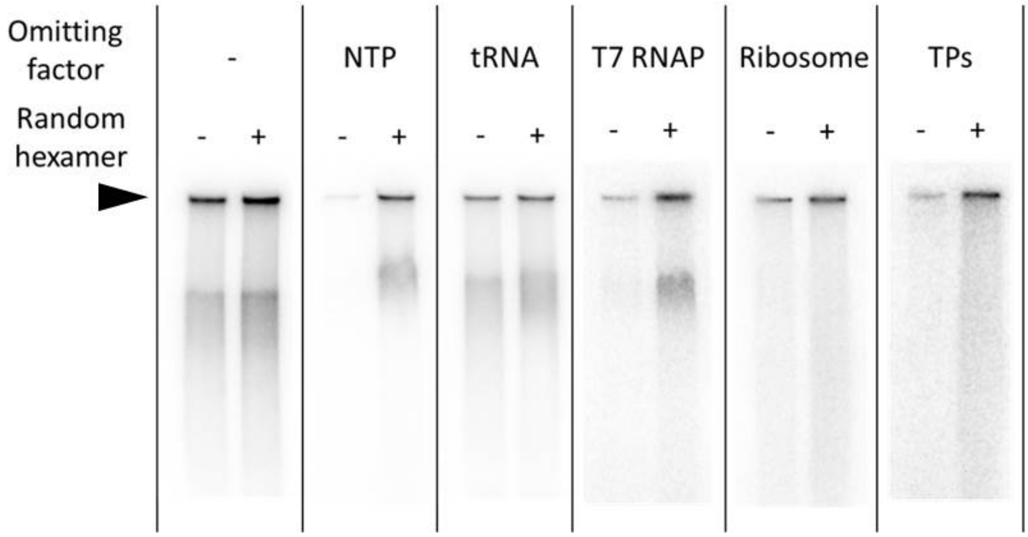

Figure S6 Whole gel images of Figure 6

The quantified bands are indicated (arrowheads).
